# Truncating leguminous fixation and post‐harvest scavenging: Two pathways for reducing nitrate leaching in potato rotations

**DOI:** 10.1002/jeq2.70304

**Published:** 2026-10-27

**Authors:** Yefang Jiang, Judith Nyiraneza, Tandra Fraser, Christine Noronha, Ana Kostic, Danielle Murnaghan, Jessica Matheson

**Affiliations:** ^1^ Charlottetown Research and Development Centre of Agriculture and Agri‐Food Canada Charlottetown Prince Edward Island Canada

## Abstract

Nitrate leaching from intensive potato production is a significant contributor to groundwater contamination and estuarine eutrophication in Atlantic Canada. This 5‐year study (2018–2023) evaluated two cover‐cropping strategies in a barley (*Hordeum vulgare* L.)–red clover (*Trifolium pratense* L.)–potato (*Solanum tuberosum* L.) rotation in Prince Edward Island: (1) mid‐season red clover termination followed by buckwheat (*Fagopyrum esculentum* Moench) seeding, and (2) winter rye (*Secale cereale* L.) established after potato harvest. Nitrate leaching was quantified using tile‐drain concentrations integrated with LEACHN‐modeled drainage. Introducing buckwheat reduced post‐potato nitrate leaching by 30.6% (*p* = 0.05) in 2019/20. The early termination of red clover truncated the biological N_2_ fixation period, fundamentally reducing the N pool available for mineralization and subsequent leaching. Conversely, winter rye acted through a traditional “Scavenging” pathway, reducing post‐harvest leaching by a pooled 37% (*p* = 0.02) through active N uptake (average 28 kg N ha^−1^). Neither strategy significantly impacted subsequent marketable potato yields. These results demonstrate that while winter rye is a robust scavenger of residual N, the early termination of leguminous forages to plant cover crops like buckwheat provides an effective “source‐control” intervention. Overall, both buckwheat and winter rye cover cropping strategies effectively mitigated nitrate leaching in this potato‐based rotation—through different mechanisms—and offered a practical framework for reducing the environmental footprint of potato production in humid, temperate regions.

## Core Ideas

- Early termination of red clover reduced post‐potato nitrate leaching by 30.6%.
- Leaching was reduced by truncating biological N fixation rather than buckwheat N uptake.
- Post‐potato winter rye reduced nitrate leaching by a pooled 37% through active scavenging.
- Neither buckwheat nor winter rye cover cropping negatively impacted subsequent potato yields.
- Managing leguminous N‐fixation is as critical as post‐harvest scavenging for mitigating nitrate loss in potato rotations.

## Plain Language Summary

Growing potatoes can cause nitrate to wash from fields into groundwater, harming water quality and wasting nitrogen. We tested two cover cropping practices in Prince Edward Island to reduce these losses: planting buckwheat after ending red clover early and planting winter rye after potato harvest. Compared with bare soil, both practices reduced nitrate leaching, especially in fall and spring when most losses occur. Buckwheat lowered post‑potato nitrate loss by about 30%, and winter rye cut losses by about 37% across years. Importantly, potato yields were not reduced. These results show that strategic cover cropping can protect water quality while maintaining crop production.

**Abbreviations**
PEI: Prince Edward Island
SOM: soil organic matter

## 1 INTRODUCTION

Nitrate leaching from intensively managed croplands poses major environmental and economic concerns, contributing to groundwater contamination, eutrophication, and the loss of valuable nitrogen (N) resources (Bijay‑Singh & Craswell, 2021; Pennino et al., 2018). When nitrate‐rich groundwater discharges into streams, a portion is converted to N_2_O through denitrification, accounting for ∼10% of global N_2_O emissions (Beaulieu et al., 2011). Leaching occurs when downward water movement exceeds evapotranspiration and the soil exceeds field capacity, particularly during precipitation, snowmelt, or irrigation events (Meisinger & Delgado, 2002). The amount of nitrate available for loss depends on interactions within soil C and N cycles (Jiang et al., 2012).

Cover crops are widely used to mitigate nitrate leaching by scavenging residual soil nitrate and reducing losses during fallow periods between cash crops (De Notaris et al., 2018; Tosti et al., 2014). Winter cover crops, particularly grasses, are effective at capturing nitrate after main crop harvest, reducing the pool susceptible to leaching during wet periods (Constantin et al., 2010; Dabney et al., 2001). The N they assimilate is later released through biomass decomposition, supplying N credits to subsequent crops (Quemada et al., 2013; West et al., 2020). Beyond nitrate reduction, cover crops also limit erosion, improve soil health, suppress weeds and pests, and enhance soil carbon (Aiyer et al., 2024; Poeplau & Don, 2015). However, their effects on soil C and N cycling can also influence nitrate leaching in less predictable ways; for example, red clover has been shown to improve soil aggregation but increase nitrate leaching relative to timothy (Whittaker et al., 2023). Understanding these interactions is particularly important in humid regions with sandy soils where leaching risk is high.

Potato production is often associated with substantial nitrate leaching due to shallow rooting and high N demand, typically resulting in 40%–60% N recovery efficiency (Stites & Kraft, 2001; Zebarth & Rosen, 2007). In Prince Edward Island (PEI), which produces ∼25% of Canada's potatoes, processing potatoes fertilized at the recommended 180 kg N ha^−1^ frequently show elevated nitrate leaching, particularly after harvest (Jiang et al., 2019, 2022). Roughly 10%–15% of wells in intensively farmed watersheds exceed the Health Canada drinking water limit of 10 mg N L^−1^ (Zebarth et al., 2015), and nitrate‐rich groundwater discharging into estuaries has contributed to eutrophication and anoxic events across >15 PEI estuaries (Jiang et al., 2015). Reducing nitrate loss from potato systems is therefore essential for protecting drinking water and aquatic ecosystems.

Potato farming in PEI commonly followed a 3‐year barley–red clover–potato rotation, where red clover is plowed down in the fall of the third year to maintain soil organic matter (SOM) (PEIAIC, 2022; Whittaker et al., 2023). However, failure to account for large N credits from red clover can lead to excessive fertilizer application, increased nitrate leaching, and reduced potato yield (Azimi et al., 2022; Jiang et al., 2019; Liang et al., 2019). Given the variability in soil N supply, residual N from previous crops, and potato N uptake, fertilizer management alone cannot eliminate leaching risk (Zebarth & Rosen, 2007). Cover crops provide a practical means to capture residual nitrate while protecting soil from erosion (Shrestha et al., 2010). Yet the short growing season following long‑season potato harvest limits cover crop establishment in PEI. Winter rye, widely used in other northern regions (Farzadfar et al., 2021; Feyereisen et al., 2006), may be promising but has been little studied in Atlantic Canada.

Wireworm damage has become a major concern in the PEI potato industry. Buckwheat used as a cover crop can suppress wireworm populations because it acts as a toxic food source during their feeding period in fall (Noronha et al., 2023). A proposed management strategy involves terminating red clover in mid‑August and planting buckwheat as a fall cover crop. Buckwheat may also reduce nitrate leaching by capturing N mineralized from terminated red clover during fall and early winter. Being winter‑killed, buckwheat is assumed to release little nitrate during late‑winter and early‑spring leaching periods because decomposition is slow in frozen soils. Furthermore, using buckwheat eliminates the need for fall primary tillage normally required for red clover, potentially reducing soil erosion (Edwards et al., 1998). However, the effect of N uptake by buckwheat on nitrate leaching has not been empirically tested. Terminating red clover in mid‐August and planting buckwheat as a fall cover crop shortened the red clover growing period, thereby reducing its N fixation and contribution to the system, which may in turn reduce nitrate leaching. This indirect mechanism, whereby cover crops mitigate nitrate leaching by reducing legume N fixation, has not been empirically evaluated.

This study evaluated nitrate leaching in the common barley–red clover–potato rotation in PEI under two cover‑cropping strategies: (1) mid‑August termination of red clover followed by buckwheat, and (2) winter rye planted after potato harvest. These were compared with conventional fall red clover plowing and bare soil. We hypothesized that buckwheat would reduce pre‐potato nitrate leaching by utilizing N released from terminated red clover and that winter rye would reduce post‐potato nitrate leaching by assimilating residual nitrate. Because the benefits of buckwheat cover cropping for wireworm suppression have been reported elsewhere (Noronha et al., 2023), and because wireworm pressure was low at the experimental site, this study focused exclusively on buckwheat's co‐benefit for nitrate leaching reduction and did not evaluate its effects on wireworm control.

## 2 MATERIALS AND METHODS

### 2.1 Field experiment

The field experiment was conducted from 2018 to 2023 at the Agriculture and Agri‐Food Canada (AAFC) Research Farm in Harrington, PEI (46.21°N, 63.90°W). The site has ∼6% slope and Orthic Humo‐Ferric Podzol soil formed from glacial till (MacDougall et al., 1988). Soil sampling in 2021 showed 3.0%, 2.8%, and 1.6% SOM at 0–0.15, 0.15–0.3, and 0.3–0.45 m (Broadbent, 1965). Texture at 0–0.15 m was 49.8% sand, 42.4% silt, and 7.8% clay (Kettler et al., 2001). Soil pH averaged 6.1 (0–0.15 m) and 5.9 (0.15–0.30 m). A uniform barley crop was planted before initiating treatments to reduce fertility heterogeneity.

The rotation began with red clover in 2018 and Russet Burbank potatoes in 2019 and 2022. All crops were rain‐fed, and local recommendations were followed for pest, disease, and weed control (PEIAIC, 2022). Fertilizer N was applied at 170 kg N ha^−1^ for potatoes and 76.5 kg N ha^−1^ for barley (PEI Government, 2014). Red clover, buckwheat, and winter rye received no N fertilizer. Cropping sequences and agronomic practices are provided in Table S1.

The experiment used a completely randomized design with three treatments, each replicated three times (nine plots) (Figure 1). Treatment 1 (T1), the control, followed conventional management: fall plow‐down of red clover, potatoes planted the next spring, and bare soil after harvest. Treatment 2 (T2) involved mid‐season termination of red clover with glyphosate and disking, followed by buckwheat cover cropping; soils were bare after potato harvest. Buckwheat was winter‐killed and incorporated the following spring. Buckwheat was intended for both 2018 and 2021 but was only established in 2018 due to COVID‐19 constraints. Treatment 3 (T3) followed conventional red clover plow‐down but planted winter rye after potato harvest (2019 and 2022). Spring 2018 soil nitrate at 0–30 cm was 33.8, 39.5, and 30.6 kg N ha^−1^ for T1–T3, respectively (*p* < 0.01; Maynard et al., 2008).

Plot size ranged from 0.27 to 0.42 ha. Each plot was drained using two 0.1‐m PVC tile lines installed at ∼0.85‐m depth and spaced 15 m apart (Jiang et al., 2011; Milburn et al., 1997). Perimeter drains prevented cross‐plot flow. Each plot had a tipping‐bucket flow meter and automated sampling station.

**Core Ideas**

- Early termination of red clover reduced post‐potato nitrate leaching by 30.6%.
- Leaching was reduced by truncating biological N fixation rather than buckwheat N uptake.
- Post‐potato winter rye reduced nitrate leaching by a pooled 37% through active scavenging.
- Neither buckwheat nor winter rye cover cropping negatively impacted subsequent potato yields.
- Managing leguminous N‐fixation is as critical as post‐harvest scavenging for mitigating nitrate loss in potato rotations.

### 2.2 Sampling and analysis

#### 2.2.1 Tile drainage, soil moisture, and temperature monitoring

Tile flow was recorded hourly using a CR10X data logger (Campbell Scientific). Automatic samplers (ISCO 6712) collected 250 mL daily samples during tile flow. Samples were stored at 3°C for ∼1 week before analysis. Nitrate, nitrite, and ammonium were analyzed using a Lachat QuikChem 8500; nitrite and ammonium were negligible and not reported. Soil moisture and temperature were monitored hourly at 0.15‐ and 0.30‐m depths in two plots using HOBO H21‐USB loggers and used to model soil drainage for leaching estimates.

#### 2.2.2 Nitrate leaching assessments

Because tile nitrate concentration alone does not represent nitrate flux, cumulative nitrate leaching was calculated for each defined leaching period and compared statistically among treatments. When tile data were available, drainage‐weighted nitrate concentrations and nitrate loads were also calculated.

Nitrate leaching primarily occurs when soil moisture exceeds field capacity, usually during heavy rain or snowmelt, and often reflects nitrate accumulated during the preceding summer (Jiang et al., 2012, 2019). Thus, leaching periods were defined using cultural records, soil drainage model outputs, tile flow, and soil moisture data. Total nitrate leaching over each rotation cycle was used to account for cross‐year effects of cover crop residue decomposition. Cumulative nitrate leaching (kg N ha^−1^) was calculated as the product of daily nitrate concentration and daily soil drainage.

Daily tile nitrate data (October 16, 2018–December 31, 2023) were interpolated using one‐way spline methods (SRS1) when missing. Tile drainage values were not used as soil drainage because (i) tile drains capture only part of total drainage, (ii) reliable tile measurements were unavailable before November 2019, and (iii) equipment failures caused additional gaps. Instead, the LEACHN model (Hutson, 2003) developed by Jiang et al. (2019, 2022) was updated to include 2018–2023 data and used to calculate daily soil drainage. Because tile spacing was wide and LEACHN cannot explicitly simulate tile drains, it was assumed that tiles did not significantly alter soil water or N cycling. Soil drainage conditions were assumed uniform across plots. The model was recalibrated within measured soil property ranges and validated using soil moisture and temperature data.

#### 2.2.3 Crop tissue and yield sampling

Crop tissue was analyzed for C and N to support interpretation of N cycling. Samples were collected from 0.5 × 0.5 m quadrats. Red clover was cut two or three times with residue left in field during the season, and above‐ground samples were collected 1 day before each cut. Buckwheat biomass was sampled in fall 2018 before senescence. Barley and rye grain and straw were sampled at harvest; additional rye samples were taken in spring 2023.

During potato years, three composite tissue samples per plot were collected before vine desiccation, each consisting of four adjacent plants. Vines and tubers were weighed, subsampled, dried at 60°C, and ground for C and N analysis using a Vario Max CN Analyzer. Field‐scale N accumulation was calculated using planting density (0.91 m row spacing, 0.38 m in‐row spacing). Tuber yields were based on three interior 8‐m rows; marketable yield was total yield minus cullage. Yield comparisons were made between buckwheat plots and control plots (2019 and 2022), and between winter rye and control plots in 2022.

For all crops, ∼1 kg subsamples were dried at 60°C and ground to 1 mm for CN analysis. Whole‐plant N for barley, rye, and buckwheat was scaled from above‐ground biomass; root N was not included (<20% of total N; Hansson et al., 1987; Patel et al., 2016). Red clover N accumulation included root N and aboveground N. Root N was estimated as 30% of the first‐cut above‐ground N (Bolinder et al., 2002; Bowren et al., 1969). Aboveground N included fixation N and recycled N from cut residues by assuming that 50% of the N in previously cut residue was mineralized and recycled into subsequent regrowth (Sarrantonio, 2012).

#### 2.2.4 Soil sampling

Soil nitrate was sampled annually in spring before planting and in fall after harvest, except in spring 2020 due to COVID‐19 restrictions. Sampling aimed to identify soil N sources contributing to nitrate leaching. In each plot, composite soil samples were taken at 0–0.15 m and 0.15–0.30 m using a 0.05‐m Dutch auger. Three composite samples per depth were collected per sampling event. Samples were air‐dried, stored at room temperature, and extracted with KCl following Maynard et al. (2008). Extracts were kept at <4°C and analyzed within 2 weeks for nitrate using a Lachat QuikChem 8500. A 10‐g subsample was dried at 105°C for ∼24 h to determine gravimetric moisture content. Soil nitrate contents for each depth were calculated using bulk densities in Jiang et al. (2011). Plot‐level values were the averages of the three composite samples. Ammonium contents were negligible and not reported.

### 2.3 Statistical analysis

#### 2.3.1 Nitrate leaching and crop yield

Nitrate leaching was analyzed separately for each rotation cycle and for three accounting periods: (i) before cover crop seeding, (ii) after cover crop seeding, and (iii) the full rotation. Pairwise comparisons were made between the control (T1) and buckwheat (T2) and between T1 and winter rye (T3); T2 and T3 were not compared because buckwheat and winter rye were established in different crop phases and the experiment was not aimed to identify which of the cover crops is better. Analyses were conducted using SAS PROC TTEST (SAS Studio 3.81). With limited replication, normality was assumed based on the stability of cumulative flux data and visual inspection of residuals. Equality of variance was assessed using the Folded *F*‐test; pooled or Satterthwaite *t*‐tests were applied based on variance homogeneity.

Long‐term effects of winter rye (across 2019 and 2022 rotations) were evaluated using a linear mixed‐effects model (SAS PROC MIXED). Fixed effects included Cycle, Treatment, and their interaction, with a random intercept for the Cycle × Treatment combination to account for inter‐annual variability. Model assumptions were assessed via residual diagnostics. Q‐Q plots showed residuals aligned with the theoretical distribution, and mixed models are robust to mild non‐normality. Residual‐versus‐predicted plots showed no evidence of heteroscedasticity, supporting the assumption of variance homogeneity.

#### 2.3.2 Soil nitrate content

Soil nitrate was analyzed using a linear fixed‐effects model (SAS PROC MIXED) with Treatment as the fixed effect. Residuals were evaluated using PROC UNIVARIATE and visual inspection of *Q*–*Q* plots and histograms. Due to low replication, graphical diagnostics were prioritized. Residual‐versus‐predicted plots indicated no heteroscedasticity.

## 3 RESULTS AND DISCUSSION

### 3.1 Weather

The region receives a mean annual precipitation of 1109 mm, about 25% of which falls as snow (2000–2023; Environment and Climate Change Canada Harrington). Growing‐season (June–September) precipitation ranged from 154 mm (2001) to 479 mm (2008), averaging 354 mm (Figure 1). Although long‐term monthly precipitation was relatively stable, interannual variability was large and can cause crop stress and variable yields (Jiang et al., 2022; 2024).

**FIGURE 1:**
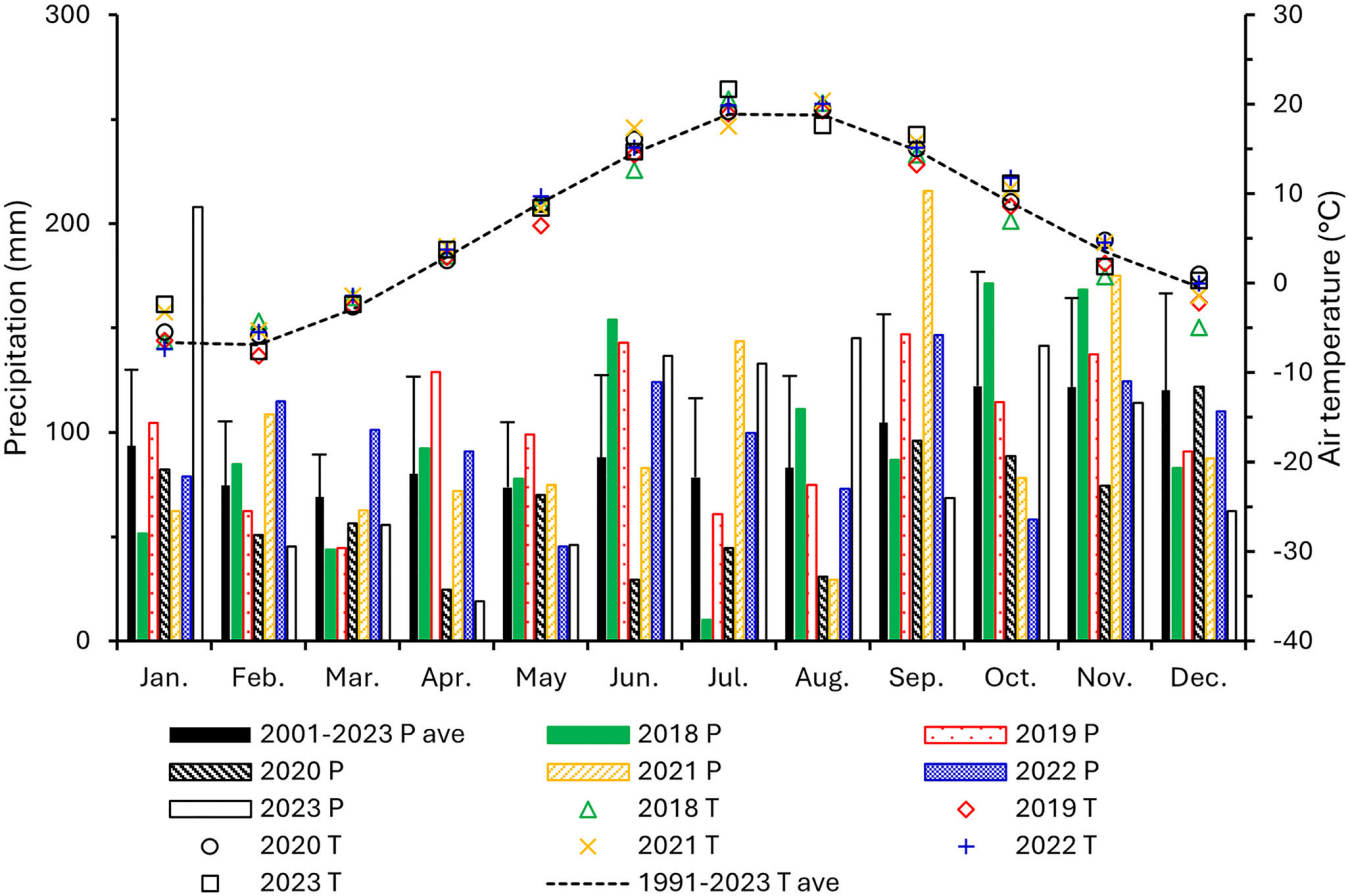
Monthly precipitation (P) and air temperature (T) (sourced from the Harrington weather station maintained by Environment and Climate Change Canada).

In 2022, precipitation was uniformly distributed and matched estimated potato evapotranspiration (353 mm; Jiang et al., 2024). Total growing‐season precipitation in 2019 was also adequate (426 mm), but high temporal variability—including a July dry spell—likely stressed the crop. The frost‐free period ranges from 100 to 160 days. Growing‐season air temperatures in 2019 and 2022 were near long‐term means, except for September 2019, which was ∼2°C cooler (Figure 1). October temperatures (5.5°C–12.9°C; mean 9°C) highlight the need for early October seeding to ensure successful winter rye establishment (Lawson et al., 2015).

### 3.2 Biomass N and crop yields

Red clover biomass and N accumulation were similar in T1 and T3 in 2018 (Table 1), as expected because management was identical before sampling. Both metrics were lower than in previous studies (Jiang et al., 2019; Liang et al., 2019) because clover was spring‐seeded in 2018 rather than under‐seeded the previous year, resulting in a shorter growth period. In T2, earlier termination for buckwheat establishment further reduced biomass and N accumulation to <46% of that in T1 and T3, consistent with previous reports showing reduced N fixation with shorter growth duration (Arlauskienė & Šarūnaitė, 2023; Coombs et al., 2017; Farzadfar et al., 2021; Yang et al., 2024).

**TABLE 1:** Plant biomass, N, and C (treatment averages).

			Grain or potato tuber biomass (dry)			Above‐ground residue biomass (dry)			
Year	Treatment	Crop	Yield (Mg ha^−1^)	N (%)	C (%)	Yield (Mg ha^−1^)	N (%)	C (%)	Total plant N (kg N ha^−1^)
2018	T1	Red clover				3.3	3.3	42.9	115 (21)^a^
2018	T2	Red clover				1.3	3.1	42.9	54 (16)^a^
2018	T2	Buckwheat				0.7	0.6	43	4.2
2018	T3	Red clover				3.6	3.5	42.7	100 (16)^a^
2019	T1	Potato	4.8	1.5	41.2	1.8	3.0	35.1	126
2019	T2	Potato	4.7	1.5	41.3	1.5	2.8	34.7	113
2019	T3	Potato	4.6	1.5	41.1	1.7	3.2	36.1	124
2020	T1	Barley	4.6	2.1	40.8	2.4	1.0	43.2	118
2020	T2	Barley	4.7	2.1	40.6	2.5	0.9	42.8	122
2020	T3	Winter rye	3.9	1.7	40.2	2.9	0.8	44.1	87
2021	T1	Red clover				6.3	2.4	42	137 (22)^a^
2021	T2	Red clover				7.5	2.4	42	159 (28)^a^
2021	T3	Red clover				2.1	3.2	43.6	68 (20)^a^
2022	T1	Potato	7.8	1.4	39.3	2.1	2.7	36.2	167
2022	T2	Potato	6.8	1.5	39.3	1.9	2.7	36.2	151
2022	T3	Potato	7.7	1.4	39.1	1.9	2.6	36.5	159
2023	T1	Barley	3.9	1.9	40.6	2.7	0.6	43.4	81
2023	T2	Barley	3.8	1.9	40.5	3	0.6	43.3	84
2023	T3	Winter rye	4.1	1.3	40.1	4.2 (2.5)^b^	0.4(1.6)^b^	44(41)^b^	81(40)^b^

*Notes*: T1, T2, and T3 represent plots of treatments 1–3. Total plant N includes measured above‐ground residue N and estimated root N in fall. Root N for barley, buckwheat, and stolon N for potato were not included.

^a^ Value in the bracket stands for the estimated root N of red clover.

^b^ Value in the bracket represents spring sampling of winter rye.

In contrast, red clover biomass and N accumulation in 2021 were higher than in 2018 for both T1 and T2 (Table 1) due to the longer growth period associated with under‐seeding and higher growing‐season rainfall in 2021 (472 mm) than in 2018 (362 mm). Because buckwheat was not planted in T2 in 2021, T1 and T2 had comparable clover productivity. Red clover in T3 accumulated substantially less biomass and N (67%–72% and 50%–57% lower, respectively), because clover was seeded after winter rye harvest in 2020 rather than under‐seeded, shortening the growing period.

Winter rye in 2020 accumulated less biomass and N than barley, but by 2023, biomass was similar between the two crops, suggesting that the low winter rye productivity in 2020 reflected extreme dry conditions rather than species differences.

Neither buckwheat nor winter rye significantly affected subsequent potato yields (Table 2). Marketable yields of 28 Mg ha^−1^ in 2019 were similar to the provincial average of ∼30 Mg ha^−1^, while yields (34 Mg ha^−1^) in 2022 were slightly higher. Potato biomass and plant N levels were higher in 2019 than in 2022, consistent with the yield differences.

### 3.3 Soil nitrate content

During the first rotation cycle (2018–2020), soil nitrate at 0–0.3 m was similar across treatments in spring and fall 2018 and fall 2019, although buckwheat plots (T2) tended to have slightly lower nitrate in fall 2019 (Figure 2). Similar values in spring 2018 were expected due to identical pre‐experiment management. No significant differences were detected by fall 2018, likely because the effects of buckwheat replacement had not yet produced measurable changes in N cycling.

**FIGURE 2:**
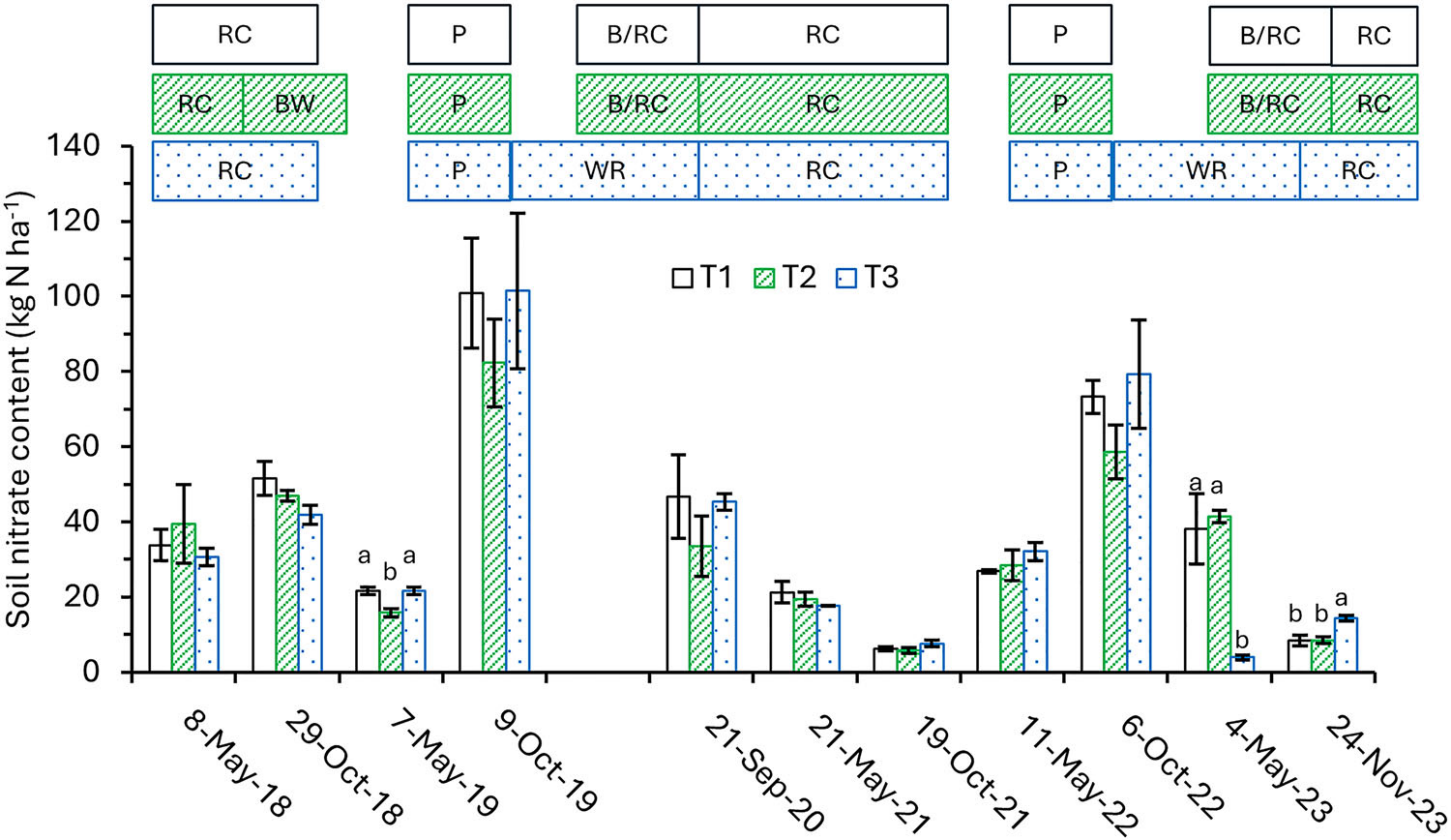
Soil nitrate content means and standard errors (0‐ to ‐0.3‐m depth) across crop phases. The rectangular boxes indicate the duration of each crop under the respective treatment. T1, Treatment 1; T2, Treatment 2; T3, Treatment 3; P, potato; RC, red clover; BW, buckwheat; B, barley; WR, winter rye.

From fall 2020 to fall 2022, no differences in soil nitrate were detected among treatments because buckwheat was not seeded in T2 in 2021 and the rye/red clover in T3 in 2020 functioned similarly as the barley/clover in N uptake in T1, making all treatments effectively similar during this period.

In spring 2023, winter rye significantly reduced soil nitrate content compared with conventional treatments (T1 + T2), consistent with biomass N results. By fall 2023, soil nitrate in T3 exceeded that in T1 and T2 because red clover seeded after rye harvest in T3 had limited growth and thus lower N uptake compared with under‐seeded red clover in T1 and T2.

### 3.4 Tile drainage and soil drainage

Tile drainage data were unavailable or unreliable before spring 2020 due to equipment repairs and were therefore excluded (Figure 3). Tile flow occurred mainly during fall, winter, and spring when precipitation or snowmelt exceeded evapotranspiration (Jiang et al., 2011) and soil moisture stayed above field capacity (Figure S2).

**FIGURE 3:**
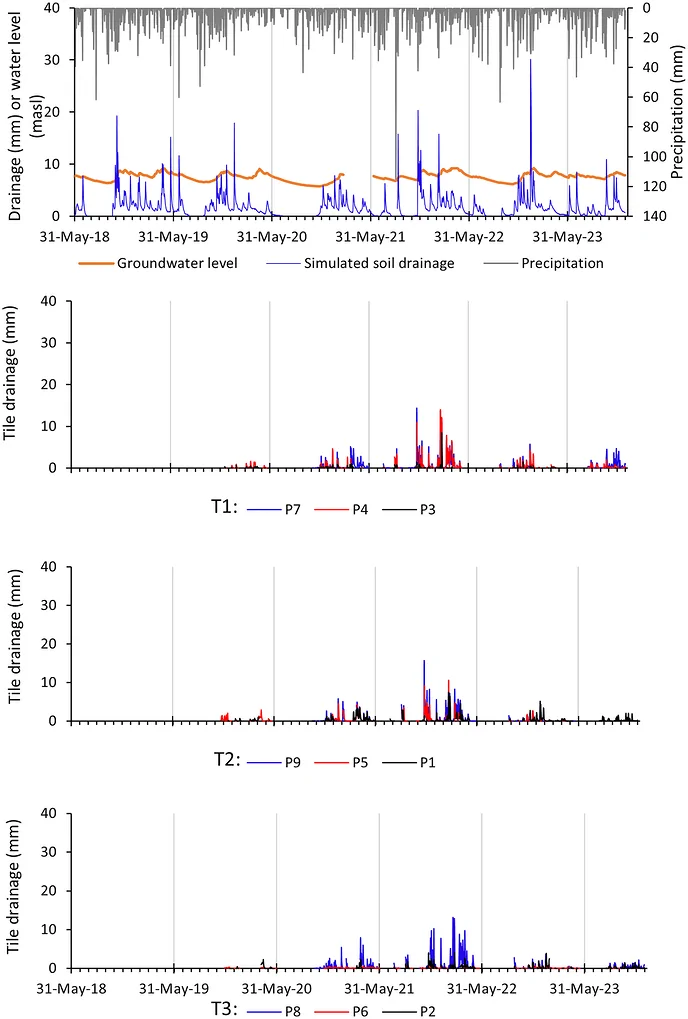
Daily precipitation at Harrington, simulated soil drainage, observed tile drainage across the experimental plots, and groundwater level. T1–T3 indicate Treatments 1–3; P1–P9 denote Plots 1–9; masl  =  meters above sea level. Groundwater level data were obtained from the Sleepy Hollow monitoring station (46°17′8″ N, 63°12′12″ W) operated by the Prince Edward Island (PEI) government.

Simulated soil drainage captured the timing of observed tile drainage and trends in the shallow groundwater table (Figure 3), indicating accurate simulation of recharge processes. However, observed tile drainage represented only 8.2%–23% of simulated soil drainage, consistent with earlier findings in this field (Burton et al., 2024; Jiang et al., 2011). Several factors explain this discrepancy: (i) The nominal plot boundaries do not reflect the true hydraulic capture zone of the drains. With ∼15 m tile spacing in the well‐drained sandy soils, a large fraction of soil drainage bypasses the tiles (Jiang et al., 2011; 2012). Comparison between simulated soil drainage and observed tile drainage for various periods is summarized in Table S3. (ii) Some tipping buckets malfunctioned, and monitoring systems were often shut down during summer, missing drainage events. (iii) Differences in tile installation, micro‐topography, and soil characteristics led to variable tile discharge among plots. For example, Plot 8 exhibited more drainage than Plot 3 despite similar treatments. Simulated annual drainage also aligned with watershed‐scale recharge estimates for the area (Crossley et al., 2025). These consistencies support the use of simulated soil drainage, rather than tile measurements, for calculating nitrate leaching (Jiang et al., 2019).

### 3.5 Tile drainage nitrate concentrations

The time series of daily tile drainage nitrate concentrations for all plots (based on 9299 samples from 2018 to 2023) are shown in Figure 4. Each point represents an individual sample; dotted spline lines interpolate between sampling dates to aid visualization of temporal changes and to estimate daily concentrations for leaching calculations. Nitrate concentration patterns were generally consistent across treatments, except for Plot 3 in T1, which consistently showed unusually low concentrations and flow volumes. Because the cause was unknown, Plot 3 was excluded from all nitrate leaching analyses.

**FIGURE 4:**
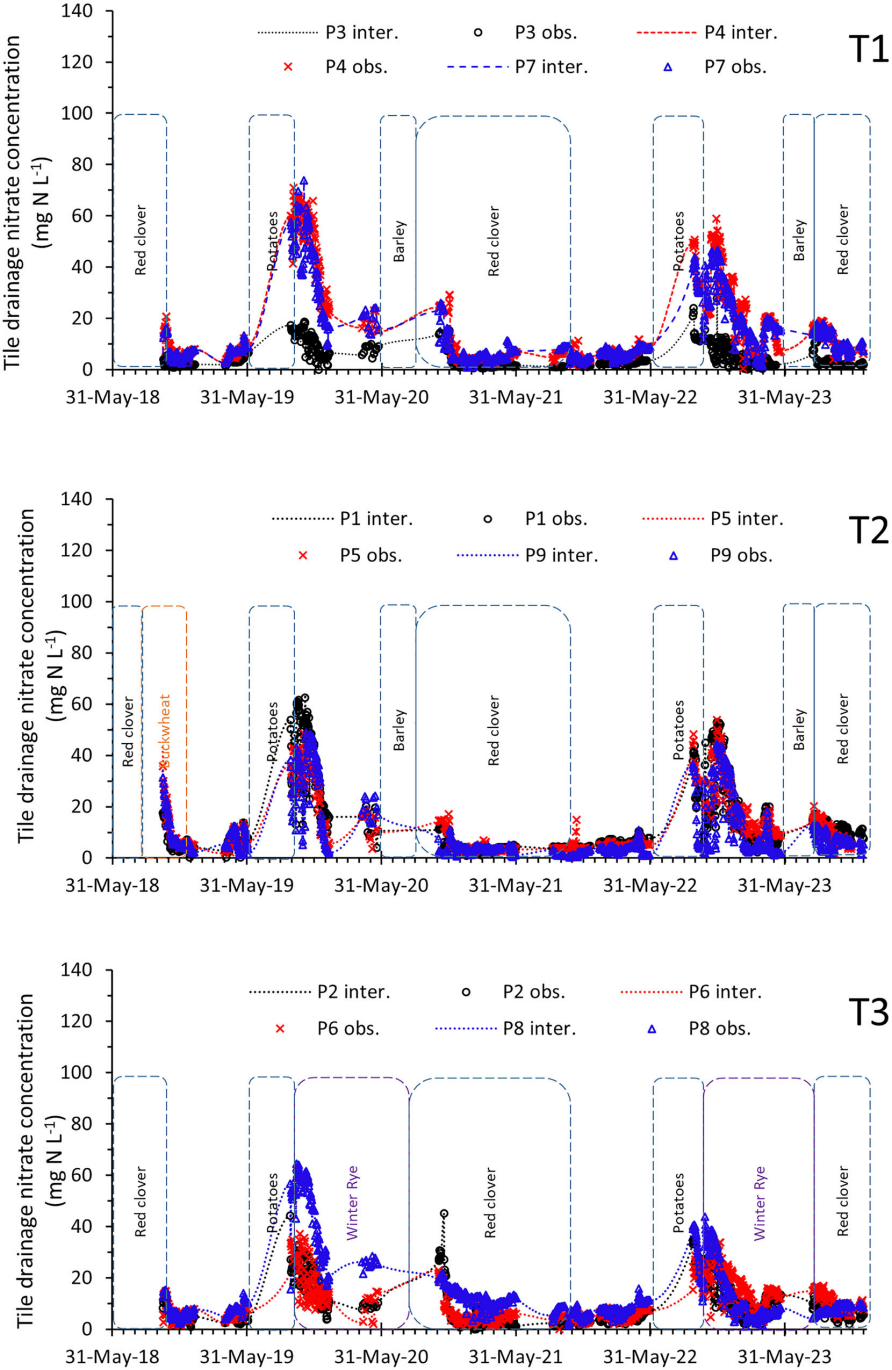
Tile drainage nitrate concentrations across treatments. T1–T3 represent Treatments 1–3. Each point corresponds to one tile‐drainage sample; dotted lines connect sample points during non‐discharge and sampling periods to illustrate temporal changes. *Inter*. and *Obs*. denote one‐way spline‐interpolated and observed concentrations. Dashed boxes outline the duration of each crop phase.

Across plots, nitrate concentrations typically peaked after crop harvest, especially following potatoes, coinciding with the onset of tile flow in fall. Concentrations then declined through winter and reached minima in late winter or early spring. These postharvest patterns resemble breakthrough curves (Freeze & Cherry, 1979), indicating that residual soil nitrate moves downward as a distinct pulse to the tile depth (0.85 m). The two post‐harvest peaks observed in 2022 likely reflect separate leaching pulses from fertilizer‐derived nitrate and nitrate released from SOM mineralization. Small fluctuations during mid‐winter and early spring likely reflected continuous, low‐level mineralization. Tile drainage nitrate concentrations generally mirrored the trends found in lysimeter sampling for the same rotation (Jiang et al., 2019, 2022) but exhibited less variability. This stabilization is due to the integrated effect of much larger catchment area of the tile drains compared to lysimeters.

Post‐potato concentrations varied from 4 to 75 mg N L^−1^, typically higher than concentrations observed during the rotational crop phases (1–45 mg N L^−1^) (Figure 4). In general, both buckwheat and winter rye reduced post‐potato harvest nitrate concentrations compared with the control. Following potato harvest in 2019, nitrate concentrations in tile drainage under the control treatment ranged from 44 to 75 mg N L^−1^ during September to October 2019 and declined to 9.9–21 mg N L^−1^ by January 2020. In comparison, concentrations under the buckwheat and rye rotations decreased from 47–62 to 3.7–5.7 mg N L^−1^ and from 56–64.4 to 4.0–8.9 mg N L^−1^, respectively, over the same period. More than 97% of samples (214 samples) collected under the control treatment exceeded the Canadian drinking water guideline of 10 mg N L^−1^, compared with approximately 90% of samples collected under the buckwheat (321 samples) and rye (322 samples) treatments. Similar nitrate concentrations in tile drainage were observed during the post‐potato period in 2022, although values were slightly lower, likely due to greater drainage volumes and the associated dilution effect during the 2022–2023 drainage season. Nevertheless, the majority of samples (>60%), including those from the cover crop treatments, still exceeded the drinking water guideline. These results indicate that although the cover cropping practices reduced nitrate leaching, additional mitigation measures, such as reducing fertilizer inputs, are necessary to further decrease nitrate losses from a drinking water protection perspective.

During the second rotation cycle (when tile flow data were adequate for flow‐weighted analyses), pre‐rye tile drainage‐weighted nitrate concentrations (June 2020–October 2022) did not differ significantly between T1 and T3 (*p*  =  0.2), but winter rye reduced post‐seeding concentrations (October 2022–May 2023) by 48.6% (*p*  =  0.006), suggesting that rye growth assimilated residual soil nitrate (Table 2). No treatment effects were detected across the full rotation cycle. However, missing or unreliable tile drainage data from mid‐2023 limited data interpretation and represent a study limitation.

**TABLE 2:** Effect of winter rye cover cropping on period‐based cumulative nitrate leaching from June 2018 to December 2023.

Source of variation	Simulated soil drainage (mm)	Observed tile drainage (mm)	Nitrate leaching (kg N ha^−1^)	Tile drainage nitrate loss (kg N ha^−1^)	Tile drainage‐weighted nitrate concentration (mg N L^−1^)
**June 2018–October 2019 (before rye cover cropping period; precipitation = 1667 mm)**					
T1 (control)	780		68.4		
T3 (rye cover cropping)	780		56.3		
Standard error			5.2		
*p* value			0.36		
**October 2019–May 2020 (after rye cover cropping period; precipitation = 610 mm)**					
T1 (control)	505		165.1		
T3 (rye cover cropping)	505		105.3		
Standard error			24		
*p* value			0.3		
**June 2018–May 2020 (first potato rotation cycle period; precipitation = 2277 mm)**					
T1 + T2 (control)	1285		233.5		
T3 (rye cover cropping)	1285		161.7		
Standard error			29		
*p* value			0.3		
**June 2020–October 2022 (before rye cover cropping period; precipitation = 2632 mm)**					
T1 + T2 (control)	1174	222	60.9	9.4	4
T3 (rye cover cropping)	1174	159	71.2	11.1	5.5
Standard error		63	12.7	4.9	0.8
*p* value		0.5	0.55	0.8	0.2
**October 2022–May 2023 (after rye cover cropping period; precipitation = 667 mm)**					
T1 + T2 (control)	502	41a	113.7a	10.7a	27.8a
T3 (rye cover cropping)	502	29b	67.4b	4.4b	14.3b
Standard error		2.7	5.3	0.4	1.7
*p* value		0.05	0.0007	<0.0001	0.006
**June 2020–December 2023 (second potato rotation cycle period; precipitation = 4023 mm)**					
T1 + T2 (control)	1988	317	201.7	24.3	7.7
T3 (rye cover cropping)	1988	215	165.4	17.7	8
Standard error		72	15.6	5.9	0.53
*p* value		0.35	0.13	0.46	0.7

*Note*: Different lowercase letters indicate a significant difference between the means of groups at *p *< 0.05.

### 3.6 Nitrate leaching and tile drainage nitrate losses

#### 3.6.1 Winter rye effects

In the first rotation cycle (June 2018—May 2020), winter rye was seeded in October 2019. As expected, pre‐seeding nitrate leaching (June 2018—October 2019) did not differ among treatments (Table 2). Although winter rye reduced post‐seeding leaching by 36.2% and whole‐cycle leaching by 31%, these differences were not statistically significant (*p*  =  0.3), likely due to limited replication. Due to COVID‐19 restrictions, rye tissue and soil nitrate data were not collected in the spring of 2020 to confirm the reduction in nitrate leaching.

In the second rotation cycle (June 2020—December 2023), T1 and T2 were grouped as the conventional treatment. Consistent with the first cycle, winter rye had no effect on pre‐seeding leaching (*p*  =  0.55) or tile‐based nitrate losses (*p*  =  0.80) (Table 2) or soil nitrate content (*p* = 0.36) (Figure 2). After rye establishment (October 2022–May 2023), however, nitrate leaching decreased by 40.7% (*p*  =  0.0007), aligned with the post‐rye tile drainage‐weighted nitrate concentration results. Additionally, post‐rye tile‐based nitrate losses and soil nitrate content were also significantly reduced by 59% (*p* < 0.0001) and 90% (*p *= 0.005), respectively.

Winter rye in T3 accumulated 40 kg N ha^−1^ by May 2023 (Table 1), closely matching the measured reduction in nitrate leaching (46.6 kg N ha^−1^) during 2022–2023. Assuming comparable uptake in 2020, rye N accumulation aligns fairly well with the observed 60 kg N ha^−1^ reduction after the 2019 rye crop. This correspondence indicates that winter rye mitigated nitrate leaching primarily by assimilating residual soil nitrate following potato harvest. Other loss pathways were minor. For instance, N_2_O emissions at this site were an order of magnitude smaller than nitrate losses via tile drainage (Burton et al., 2024). In addition, fertilizers were band‐applied; therefore, ammonia volatilization was assumed to be minimal. Tile‐based nitrate losses represented 6.5%–15.6% of the model‐estimated nitrate leaching because the drain capture zone was smaller than the nominal plot area.

Pooling rotation cycles provided additional insight. Pre‐rye leaching did not differ between 2019 and 2022, despite lower growing‐season rainfall in 2019. In contrast, post‐rye leaching was 50% higher in 2019 (135 kg N ha^−1^) than in 2022 (90 kg N ha^−1^) (*p*  =  0.04), even though leaching‐season precipitation was similar. This highlights how precipitation timing shapes nitrate cycling. Across both cycles, pre‐seeding leaching did not differ between treatments, whereas post‐seeding leaching under the control was 63% higher than under rye (*p* = 0.02), reinforcing the within‐cycle patterns.

Winter rye therefore showed a consistent nitrate‐mitigation effect only during the post‐potato period, when it had access to residual soil nitrate. Rye N uptake and lower spring soil nitrate content in T3 (Figure 2) confirm this mechanism. Because rye was seeded without post‐harvest tillage, tillage effects were negligible.

These findings align with global evidence showing that non‐legume cover crops commonly reduce nitrate leaching by 40%–93% (mean 69%) in humid, sandy‐soil regions (Nouri et al., 2022), consistent with reductions reported in lettuce systems (Heinrich et al., 2014) and other North American and European studies (Fernando, 1996; Dabney et al., 2001; Tonitto et al., 2006; Constantin et al., 2010; Nyiraneza et al., 2020). The ∼37% reduction observed here falls within this established range.

Across the full rotation, cumulative nitrate leaching correlated with N surplus, which was calculated as cumulative N inputs (fertilizer plus estimated red clover fixation) minus N outputs (crop harvest) from 2018 to 2022 (Figure 5; Table S4). Although the regression is not qualified for prediction due to limited data points, it indicates the combined effects of fertilizer inputs and red clover–derived N. Shortening the clover growth period in rye plots reduced N inputs by 67–83 kg N ha^−1^, but this effect was likely modest in the short term because clover‐derived N mineralizes gradually over multiple years (Liang et al., 2019; Wyngaarden et al., 2015). Rye grain harvest also exported 66.3 kg N ha^−1^ in 2019–2020, further contributing to lower nitrate leaching. Few studies evaluate cover‐crop effects across an entire rotation; the multiyear results here underscore the importance of doing so.

**FIGURE 5:**
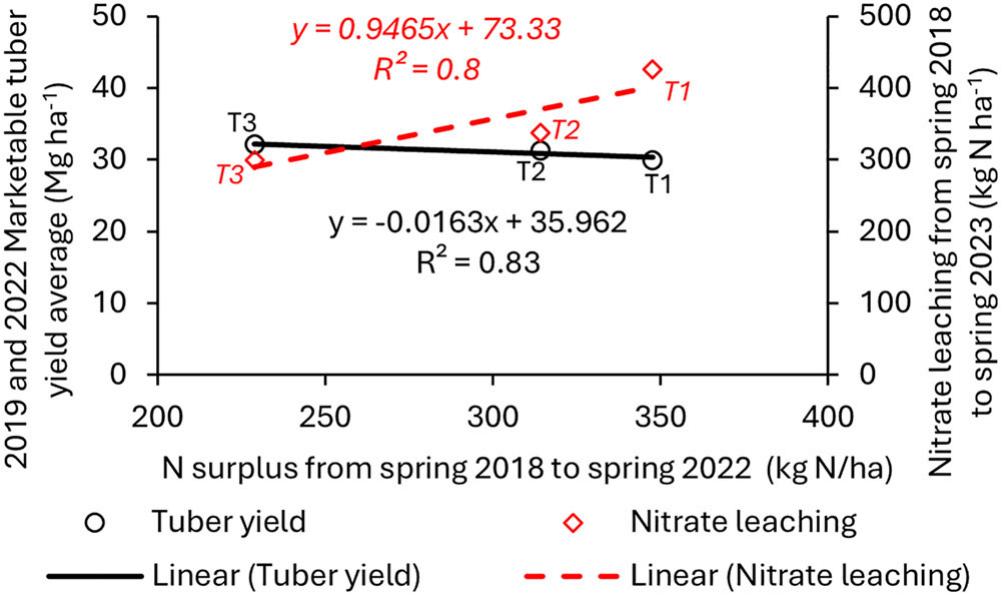
Correlations between N surplus and nitrate leaching (2018–2022) and potato tuber yield.

#### 3.6.2 Buckwheat effects

Buckwheat effects were evaluated during the first rotation cycle (June 2018—May 2020). Because no leaching occurred before buckwheat establishment in August 2018, the post‐buckwheat period was defined as June 2018—May 2019 with lag time included. For analysis, T1 and T3 were combined as the conventional treatment (Plot 3 excluded).

Buckwheat uptake did not significantly reduce nitrate leaching (August 2018—May 2019) (*p* = 0.25) (Figure 6), indicating it did not substantially immobilize nitrate released from the terminated red clover and SOM. Its N uptake was only 4.2 kg N ha^−1^ (Table 1), far too small to offset the 40.2 kg N ha^−1^ leached in conventional plots over the same period, and fall soil nitrate did not differ from the control. Glyphosate termination and disking prior to buckwheat seeding were unlikely to confound results and may even have facilitated buckwheat growth. Overall, buckwheat uptake did not meaningfully reduce residual soil nitrate.

**FIGURE 6:**
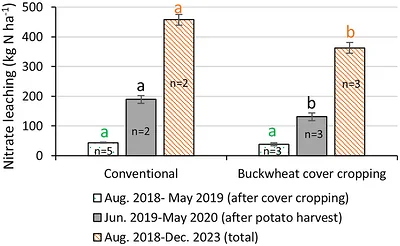
Mean nitrate leaching and standard errors under conventional (control) and buckwheat cover‐cropping treatments during the first potato rotation cycle (June 2018–May 2020). Different lowercase letters indicate significant differences between group means at *p *< 0.1; *n* stands for sample number.

Despite the limited N uptake, buckwheat significantly reduced post‐potato nitrate leaching in June 2019—May 2020 by 30.6% (*p *< 0.1) (Figure 6) and lowered soil nitrate concentrations in spring 2019 (*p *< 0.05) and, numerically, in fall 2019 (Figure 2). This reduction resulted from the shortened red clover growth period in 2018: early termination restricted biological N fixation, and clover N accumulation in T2 was < 46% of that in the control (Table 1). Reduced legume‐derived inputs led to lower mineralization and subsequently less nitrate available for leaching. Although based on a single rotation cycle, this mechanism was supported consistently by the integration of high‐frequency (i.e., daily) nitrate leaching time series of a 3‐year rotation cycle, seasonal soil nitrate, and biomass N data.

Previous work has shown that reducing red clover growth duration decreases N accumulation (Arlauskienė & Šarūnaitė, 2023; Coombs et al., 2017; Farzadfar et al., 2021; Yang et al., 2024), but few studies have linked shorter clover duration to lower nitrate leaching. Unlike typical cover‐crop mitigation, which operates through direct N uptake (Dabney et al., 2001; Nyiraneza et al., 2020), buckwheat reduced leaching indirectly by limiting red‐clover‐fixed N inputs. This suggests that buckwheat's ability to reduce nitrate leaching depends strongly on the preceding crop: benefits are likely substantial only when following a forage legume. The findings highlight the importance of considering N cycling across the entire rotation, not only during post‐harvest periods.

#### 3.6.3 Correlation between fall soil nitrate content and leaching

Residual fall soil nitrate content closely reflected subsequent nitrate leaching (Figure 7). Across years and treatments, fall nitrate content explained 65.5% of corresponding fall–winter–spring leaching (*p* < 0.001), indicating that fall soil nitrate is a strong predictor of leaching risk in this system. This relationship is consistent with earlier regional studies showing that fall residual nitrate is the major source of nitrate leaching (Jiang et al., 2011; Milburn et al., 1990; Zebarth et al., 2015). The remaining unexplained variation (∼34%) likely reflects nitrate leaching before fall soil sampling, mineralization, denitrification, nitrate transport lag times, and measurement uncertainties.

**FIGURE 7:**
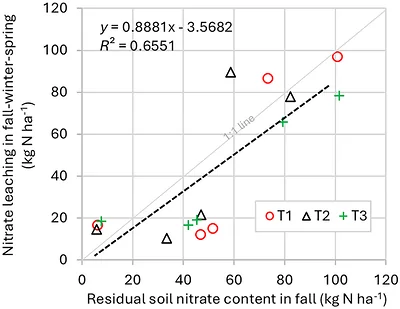
Correlation between fall soil nitrate content and nitrate leaching during the subsequent fall–winter–spring period (2018–2023). Each point represents a treatment‐level mean for a leaching period (October 16 2018—January 9 2019; September 27 2019—January 10 2020; November 3 2020–February 28 2021; September 8 2021—February 28 2022; September 24 2022—March 31 2023) paired with the corresponding fall soil nitrate content.

### 3.7 Implications for potato rotation management and limitations

The barley–red clover–potato rotation exhibited a large N surplus, with red clover serving as an important N contributor (Figure 5). This surplus likely explains the similar productivity of unfertilized rye and fertilized barley. Nitrogen surplus was negatively correlated with potato yield but positively correlated with nitrate leaching (Figure 5), indicating that excessive N inputs reduce yield while increasing losses, consistent with previous findings (Azimi et al., 2022; Jiang et al., 2019, 2022; Whittaker et al., 2023). Current N recommendations for Russet Burbank (≥170 kg N ha^−1^) underestimate clover‐derived N inputs, underscoring the need to better account for legume contributions in N management.

Replacing red clover with non‐legume forages such as timothy can reduce leaching but may compromise soil structure (Whittaker et al., 2023). Alternatively, red clover can be retained if fertilizer N inputs are reduced or if cover crops such as winter rye are used to scavenge residual nitrate. Buckwheat, although not effective at direct nitrate capture, indirectly reduced leaching by limiting clover N contributions and provided additional benefits such as erosion protection (due to reduced fall tillage) and pest suppression (Noronha et al., 2023). Because buckwheat reduces legume‐derived N inputs, its use may reduce the nitrate available for rye uptake when the two crops are combined—a system not tested in this study. Future research should evaluate combined buckwheat‐rye systems and their economic implications.

Atmospheric N deposition (<10 kg N ha^−1^; Jiang et al., 2015) and N_2_O emissions from potatoes (<3 kg N ha^−1^; Burton et al., 2024) were minor relative to nitrate leaching, and thus neglecting these terms in the N balance analysis should not materially affect the N surplus conclusions.

Three plots per treatment represent the minimum level of replication typically used in field experiments. Although the integration of long‐term, high‐frequency time‐series data on tile drainage nitrate concentrations strengthened the robustness of the nitrate leaching assessment, additional replication beyond three plots would further increase the reliability of the findings. Furthermore, the disruption of buckwheat establishment in 2022 due to COVID‐19 restrictions meant that conclusions regarding buckwheat were based on a single rotation cycle with only three plots, representing an additional limitation of this study.

## 4 CONCLUSIONS

Nitrate leaching in the barley–red clover–potato rotation occurred mainly during fall–spring, with the greatest losses following potato harvest. Leaching was strongly linked to fall soil nitrate, indicating that residual nitrate was the primary source. N surplus analysis further identified red clover as a major driver of post‐potato nitrate losses.

The early red clover termination–buckwheat management strategy did not reduce nitrate leaching during buckwheat's growth but reduced post‐potato leaching by 30.6% (*p* < 0.1) by shortening the red clover growth period and lowering N inputs, with no adverse effects on potato yield. Winter rye reduced post‐potato nitrate leaching by 36.2% (*p* = 0.3) in 2019–2020 and by 40.7% (*p* = 0.0007) in 2022–2023 by assimilating residual nitrate, and by 37% when both cycles were pooled (*p* = 0.02), also without affecting potato yield. These findings were corroborated by soil nitrate, tissue N, tile drainage, and tile drainage‐weighted concentration data.

Overall, both buckwheat and winter rye cover cropping strategies effectively mitigated nitrate leaching in this potato‐based rotation—through different mechanisms—and represent valuable tools for improving N management while maintaining crop productivity.

## AUTHOR CONTRIBUTIONS

**Yefang Jiang**: Conceptualization; data curation; formal analysis; funding acquisition; investigation; methodology; project administration; resources; software; supervision; validation; visualization; writing—original draft; writing—review and editing. **Judith Nyiraneza**: Conceptualization; writing—review and editing. **Tandra Fraser**: Writing—review and editing. **Christine Noronha**: Conceptualization; writing—review and editing. **Ana Kostic**: Data curation; investigation; methodology; writing—review and editing. **Danielle Murnaghan**: Data curation; investigation; methodology. **Jessica Matheson**: Data curation; writing—review and editing.

## CONFLICT OF INTEREST STATEMENT

The authors declare no conflicts of interest.

## Supporting information



Supplementary Material
