# Supplementary material for: Truncating leguminous fixation and post‐harvest scavenging: Two pathways for reducing nitrate leaching in potato rotations
Source: J Environ Qual. 2026 Oct 27;55(6):e70304. doi: 10.1002/jeq2.70304 (PMC13714329; doi:10.1002/jeq2.70304)
Supplement: Supplementary file 1 [file JEQ2-55-0-s001.docx]

**SUPPLEMENTAL MATERIAL**

Supplemental Fig. 1 Experimental units and tile drain layout

Supplemental Fig. 2 Daily soil moisture in the experimental field (Obs. – observations; Sim. – simulations; P3 and P4 stand for Plots 3 and 4)

Soil moisture was generally low during the growing season and high during fall–spring (Supplemental Fig. 2). During non‑growing months, soil moisture frequently exceeded the 27% field capacity for this soil type, producing gravity‑driven drainage. Simulated soil moisture closely matched observations. Occasional extreme values—very low winter readings in 2019 or abnormal readings at 0.15–0.3 m in 2021–2023—were attributed to moisture sensor malfunction in frozen soils, as expected for this sensor type.

Supplemental Fig. 3 Daily air temperature at Harrington and soil temperature in the experimental field (Obs. – observations; Sim.-LEACHN simulations; P3 and P4 stand for Plots 3 and 4)

Soil temperature tracked air temperature with smaller diurnal variation (Supplemental Fig. 3). Winter soil temperatures fell slightly below freezing, and values were similar at 0.15 and 0.3 m depths. Spatial differences between monitoring stations were minimal, and LEACHN simulations reproduced observed soil temperature patterns well.

Supplemental Table 1 Cultural practices (T1: Plots 3, 4 and 7; T2: Plots 1,5 and 9; T3 : Plots 2, 6 and 8)

| Season/year | Cultural practices |
| --- | --- |
| –2017 | The field was planted with barley–red clover–potato rotation under rainfed production. The red clover was under-seeded into the barley, and 2017 was a potato year. |
| Spring 2018 | May 18: Seeded AC Endure red clover (incorporated) at 15 kg ha^-1^ without fertilizer applied. |
| Summer 2018 | Jul. 27: Flailed the red clover on all nine plots with residue left in the field.  Jul. 31: Sprayed the red clover in the T2 plots with Roundup.  Aug. 14: Disced the T2 plots.  Aug. 16: Planted ManCan buckwheat at 75 kg ha^-1^ in the T2 plots.  Aug. 22: Flailed the red clover in the T1 and T3 plots with residue left in the field. |
| 2018 fall | Oct. 2: Clipped red clover in the T1 and T3 plots with residue left in the field.  Nov. 12: Plowed the T1 and T3 plots (mouldboard) without terminating the red clover. |
| Spring 2019 | May 8: Plowed the buckwheat (T2) plots (mouldboard).  Jun. 4: Disced all plots.  Jun.12–13: Planted Russet Burbank potatoes in all plots, and banded 1000 kg ha^-1^of 17–17–17 fertilizer mix during planting. |
| Summer 2019 | Jul. 26: Hilled potatoes.  The potatoes were managed following local standard cultural practices. |
| Fall 2019 | Sep. 19: Topkilled potatoes.  Oct. 7: Harvested potatoes.  Oct. 11: Planted A.S. Haglet winter rye at 168 kg ha^-1^ as a cover crop in the T3 plots. |
| Spring 2020 | Early Jun.: Planted Common leader barley at 185 kg/ha in the T1 and T2 plots, under-seeded with red clover at 14 kg ha^-1^, and applied 450 kg ha^-1^ of 17–17–17 fertilizer mix. |
| Summer 2020 | Aug. 10: Harvested winter rye from the T3 plots.  Aug. 19: Harvested barley from the T1 and T2 plots. |
| Fall 2020 | Sep. 30: The T3 plots were no-till seeded with Wildcat red clover at 18 kg ha^-1^ without fertilizer application.  Red clover regrew in the T1 and T2 plots. |
| Spring 2021 | May 25: No-tilled reseeded Common red clover in the T3 plots at 14 kg ha^-1^ because the red clover did not establish well. The under-seeded red clover in the T1 and T2 plots regrew. |
| Summer 2021 | Jun. 22: Flailed the red clover in the T1 and T2 plots with residue left in the field.  Aug. 10: Flailed the red clover in the T1, T2, and T3 plots with residue left in the field.  The red clover in the T2 plots should be sprayed, and the plots should be disced, and planted with buckwheat as a cover crop, as in summer 2018, but this was not implemented. |
| Fall 2021 | Nov. 8: Plowed all plots (mouldboard) without terminating the red clover. |
| Spring 2022 | May 19–20: Planted Russet Burbank potatoes in all plots, and banded 1000 kg ha^-1^ of 17–17–17 fertilizer during planting. |
| Summer 2022 | Late Jun.: Hilled potatoes.  The potatoes were managed following local standard cultural practices. |
| Fall 2022 | Oct.4: Harvested potatoes.  Oct.5: Seeded A.S. Haglet winter rye at 120 kg ha^-1^ in the T3 plots. |
| Spring 2023 | May 12: Planted Common leader barley at 185 kg ha^-1^ underseeded with Global red clover at 14 kg ha^-1^ in T1 and T3 plots and applied 450 kg ha^-1^ of 17-17-17 fertilizer mix during planting. |
| Summer 2023 | Aug.16: Harvested winter rye in the T3 plots.  Aug. 22: Harvested barley in the T1 and T2 plots. |
| Fall 2023 | Date: Seeded Wildcat red clover in the T3 plots. The red clover did not establish well.  Red clover regrew after barley harvest in the T1 and T2 plots. |
| 2024 | May 8: No-till reseeded Sanfa Fe red clover in the T3 plots at 16 kg ha^-1^. |

Supplemental Table 2 Effects of cover cropping on subsequent potato tuber yields

| Source of variation | 2019 total yield (Mg ha^-1^) | 2019 marketable yield (Mg ha^-1^) | 2022 total yield (Mg ha^-1^) | 2022 marketable yield (Mg ha^-1^) |
| --- | --- | --- | --- | --- |
| 2018 control | T1+T3 = 28.3 | 27 | T1 = 37 | T1 = 35.3 |
| 2018 buckwheat cover cropping | T2 = 30.2 | 28.7 | T2 = 34.9 | T2 = 33.2 |
| Standard error | 2 | 2 | 0.75 | 0.8 |
| *p* value | 0.46 | 0.53 | 0.17 | 0.24 |
| 2019 control |  |  | T1 = 37 | T1 = 35.3 |
| 2019 rye cover cropping |  |  | T3 = 37.8 | T3 = 36.1 |
| Standard error |  |  | 1.3 | 1.4 |
| *p* value |  |  | 0.67 | 0.69 |

Notes: T1 to T3 stand for the plots of Treatments 1 to 3. T1+T3 represents that the data from T1 and T3 were grouped.

Supplementary Table 3 Observed tile drainage, simulated soil drainage, and precipitation (mm)

| Period | T1 Ave. | T1 Stdev. | T2 Ave. | T2 Stdev. | T3 Ave. | T3 Stdev. | Ave. | Simulated | Precipitation |
| --- | --- | --- | --- | --- | --- | --- | --- | --- | --- |
| Jun/1/2018 to Oct/9/2019 |  |  |  |  |  |  |  |  | 1667 |
| Oct/10/2019 to May/31/2020 |  |  |  |  |  |  |  |  | 610 |
| Jun/1/2020 to 1/Oct/2020 |  |  |  |  |  |  |  | 1174 | 222 |
| 1/Oct/2020 to 31/May/2021 | 72.8 | 45.9 | 89.7 | 14.2 | 49.3 | 50.3 | 70.6 | 420.2 | 665 |
| 23/Jul/2021 to 31/May/2022 | 197.9 | 19.2 | 144.0 | 70.3 | 122.8 | 120.8 | 154.9 | 650.9 | 1034 |
| 23/Sep/2022 to 31/May/2023 | 47.3 | 3.7 | 43.1 | 12.3 | 36.2 | 6.4 | 42.2 | 510.0 | 797 |
| 15/Aug/2023 to 31/Dec/2023 | 52.2 | 41.0 | 23.9 | 20.7 | 30.0 | 19.4 | 35.4 | 224.0 | 460 |

Supplemental Table 4 Comparison of N surplus and nitrate leaching from 2018 to 2022 (kg ha^-1^)

| Year | Crop | T1 | T2 | T3 | Explanation |
| --- | --- | --- | --- | --- | --- |
| 2018 | Red clover/buckwheat | 94 | 38 | 84 | Fixation N input |
| 2019 | Potato | 170 | 170 | 170 | Fertilize N input |
| 2019 | Potato | -72 | -70.5 | -69 | Harvest N output |
| 2020 | Barley/Winter rye | 76.5 | 76.5 | 0 | Fertilizer N input |
| 2020 | Barley/Winter rye | -96.6 | -98.7 | -66.3 | Harvest N output |
| 2021 | Red clover | 115 | 131 | 48 | Fixation N input |
| 2022 | Potato | 170 | 170 | 170 | Fertilizer N input |
| 2022 | Potato | -109.2 | -102 | -107.8 | Harvest N output |
| Nitrogen surplus for spring 2018 – spring 2022 |  | 347.7 | 314.3 | 228.9 |  |
| Nitrate leaching for spring 2018 – spring 2023 |  | 426 | 338 | 299.2 |  |

Note: T1 to T3 stand for the plots of Treatments 1 to 3.
